# The risks of AI-generated health advice

**DOI:** 10.1016/j.eclinm.2026.103851

**Published:** 2026-03-25

**Authors:** eClinicalMedicine

As artificial intelligence (AI) becomes more embedded in daily life, growing numbers of people are turning to generative AI chatbots for health advice. OpenAI, the developer of the popular platform ChatGPT, reports that, worldwide, over 230 million people use the tool for health and wellness advice each week. In the context of overstretched health systems, online living, and convenience culture, generative AI can appear to be an accessible alternative to professional care. However, mounting evidence shows that these tools often provide misleading or dangerous information, underscoring the need for research, regulation, and public guidance.

Generative AI produces information that appears factual but may have a high risk of being inaccurate. In the context of a user asking a chatbot for medical advice, this can be dangerous. A recent study evaluating four major chatbots (Claude, Gemini, ChatGPT, and Llama) identified multiple failures in answering diagnostic or treatment questions. This included omitting safety information, such as emergency precautions related to miscarriage, and including unsafe advice, such as recommending that infants drink water. The chatbots also offered inappropriate reassurance, for example, suggesting heartburn was likely to be benign without considering patient history. Failings also occur in AI-generated search summaries. An investigation by *The Guardian* found that Google AI Overviews provided inaccurate and potentially harmful information on topics including pancreatic cancer and liver function tests. Such errors reflect absence of the clinical training and contextual reasoning that underpin medical practice and raise concerns about large-scale dissemination of health misinformation.

Mental health is an area of particular concern, with AI chatbots increasingly filling gaps in inaccessible or overstretched services. Although global statistics are lacking, surveys suggest over a third of adults in the UK and USA have used a chatbot for mental health support. The appeal is evident: no waitlists, no stigma, 24/7 availability, and, often, no cost. In settings where mental health care is scarce or stigma deters help seeking, chatbots are particularly attractive. In China, amid rising rates of mental illness, young people are turning to tools such as DeepSeek. In Nigeria, where mental health services are scarce and dismantlement of USAID has reduced external support, nonprofit initiatives are using AI to provide emotional assistance and link people to care. However, evidence suggests these tools should be used with caution. A study comparing licensed therapists with seven major chatbots found that chatbots performed poorly when responding to suicidal ideation, offering open-ended comments and failing to connect users to crisis resources.

Although AI models can perform well on certain medical benchmarks, such as achieving passing scores on standardised medical examinations, their performance in clinical decision support is currently poor. In a recent trial, chatbots given medical scenarios achieved 95% diagnostic accuracy when operating independently. However, members of the public using chatbots to assist with the same medical scenarios identified conditions in only 35% of cases. This discrepancy highlights the gap in performance between controlled testing and real-world implementation. Several factors help explain why generative AI often gives poor health advice. Models can reproduce or amplify false information (so-called hallucination). A study that embedded fabricated information in clinical prompts to induce hallucination found that major models hallucinated in 50–83% of cases, depending on the model, mentioning non-existent laboratory tests or diseases. Structural human biases can also be encoded into AI. In simulated AI–patient interactions, chatbots provided more attentive care to older and wealthier patients, recommending more tests and medications. Tackling these problems in model development, training, and regulation should be a priority to reduce risk.

As AI becomes more integrated into health care, specialist chat tools are emerging, which could mitigate some of the issues seen in generic AI models. In mental health, therapy chatbots, such as those from Wysa and Therabot, are popular, with Wysa claiming over 6 million users and counting. A randomised controlled trial of Therabot found reductions in symptoms of depression, anxiety, and eating disorders compared with a waitlist control, although the tool was not compared with standard therapy, limiting interpretation. In January, OpenAI launched ChatGPT Health, which integrates medical records and wellness-app data to provide personalised guidance. Although framed as a support tool rather than a substitute for clinical care, the tool is designed to guide “how urgently to encourage follow-ups with a clinician” and how to “prioritise safety in moments that matter”. However, an independent safety evaluation found that ChatGPT Health under-triaged 52% of emergency medical scenarios presented. Cases involving asthma exacerbation, diabetic ketoacidosis, and respiratory failure were often misclassified as suitable for delayed evaluation, and suicide-related prompts triggered inconsistent crisis-intervention responses. Anchoring bias was also observed: when family or friends minimised symptoms, the model's triage recommendations shifted to less urgent levels. These results have raised alarm over its potential for precluding life-saving treatment.

Although health authorities, such as WHO and the UK National Health Service, are experimenting with introducing AI tools, the private sector is firmly taking the lead on AI technology. This dominance gives profit-driven private companies the potential for substantial influence over global health behaviour. Yet regulation to protect the public has not kept pace. Although purpose-built AI health tools are typically regulated as medical devices, general-purpose platforms like ChatGPT fall outside traditional regulatory frameworks. Accountability is also unclear, as laws surrounding medical liability in AI are still evolving. Recent AI deregulatory measures in the USA, where many AI companies are based, are likely to delay development of regulatory laws aimed at protecting users. A global perspective in AI regulation will be especially vital in protecting users in low-income and middle-income countries, where Government AI readiness is often lagging behind high-income countries. The 2025 launch of two UN initiatives to promote international cooperation in AI governance marks progress in global efforts to harness AI while addressing the risks.

With the rapidly expanding capabilities of AI, chatbots and other AI tools have the potential to support patients and assist established health systems. However, the current risks of widely accessible, unregulated, and unsafe AI technology are profound. Safeguarding people will require coordinated global action to ensure that these technologies support, rather than undermine, safe, equitable, and trustworthy health care. While we await the technological development and regulation that will result in safer AI tools, public health messaging, for example, publicity campaigns and implementation of disclaimers, is urgently needed to raise awareness of dangers and guide safe use.

